# Increasing Access to Farmers Markets for Beneficiaries of Nutrition Assistance: Evaluation of the Farmers Market Access Project

**DOI:** 10.5888/pcd10.130121

**Published:** 2013-10-03

**Authors:** Kate Cole, Molly McNees, Karen Kinney, Kari Fisher, James W. Krieger

**Affiliations:** Author Affiliations: Kate Cole, University of Washington School of Public Health, Seattle, Washington; Karen Kinney, King County Department of Natural Resources and Parks, Seattle, Washington; Kari Fisher, Public Health–Seattle & King County, Seattle, Washington; James W. Krieger, Public Health–Seattle & King County, and the Schools of Medicine and Public Health, University of Washington, Seattle, Washington.

## Abstract

**Introduction:**

Increased acceptance of nutrition benefits at farmers markets could improve access to nutritious foods for low-income shoppers. The objective of this study was to evaluate a pilot project to increase participation by farmers markets and their vendors in the Supplemental Nutrition Assistance Program (SNAP) and Special Supplemental Nutrition Program for Women, Infants, and Children (WIC).

**Methods:**

The intervention targeted 9 markets in lower-income regions of King County, Washington. Markets and vendors were offered subsidized electronic benefits transfer (EBT) terminals for processing SNAP, and vendors could apply to accept WIC cash value vouchers. WIC staff received information on using SNAP and vouchers at farmers markets. We used mixed methods post-implementation to measure participation, describe factors in acceptance of benefits, and assess information needs for WIC staff to conduct effective outreach.

**Results:**

Of approximately 88 WIC-eligible vendors, 38 agreed to accept vouchers. Ten of 125 vendors installed an EBT terminal, and 6 markets installed a central market terminal. The number of market stalls accepting SNAP increased from 80 to 143, an increase of 79%. Participating vendors wanted to provide access to SNAP and WIC shoppers, although redemption rates were low. Some WIC staff members were unfamiliar with markets, which hindered outreach.

**Conclusion:**

Vendors and markets value low-income shoppers and, when offered support, will take on some inconvenience to serve them. To improve participation and sustainability, we recommend ongoing subsidies and streamlined procedures better suited to meet markets’ capabilities. Low EBT redemption rates at farmers markets suggest a need for more outreach to low-income shoppers and relationship building with WIC staff.

## Introduction

The lack of affordable sources of fresh produce contributes to poor nutrition in many low-income neighborhoods (1,2). To address this problem, the Centers for Disease Control and Prevention (CDC) and the US Department of Agriculture (USDA) recommend increasing access to farmers markets, which, because of their flexibility, can bring produce directly into underserved communities (3,4).

The USDA’s Supplemental Nutrition Assistance Program (SNAP) and Special Supplemental Nutrition Program for Women, Infants, and Children (WIC) allow for use of benefits at farmers markets. In 1994, USDA began issuing SNAP benefits through electronic benefits transfer (EBT) cards rather than paper coupons. Although USDA provided EBT card-reading terminals to retailers, they required electricity and a landline, which are not available at most farmers markets. Between 1993 and 2007, SNAP sales at farmers markets decreased from $9.5 million to $1.7 million nationally (5). More recently, wireless terminals have been introduced, but they remain inaccessible for many markets and vendors because of an estimated national cost of $850 per terminal, plus $40 monthly fees (5). In 2009, WIC launched cash value vouchers (in paper format) for the purchase of fruits and vegetables. Most paper vouchers are redeemed at supermarkets; states can approve vouchers for use at farmers markets, although few have done so (6).

In 2010, CDC funded Public Health–Seattle & King County through Communities Putting Prevention to Work (CPPW), a national initiative to prevent chronic disease through policy, systems, and environmental changes (7). Public Health–Seattle & King County funded the Farmers Market Access Project (FMAP). FMAP subsidized EBT terminals and facilitated WIC-authorized vendors’ participation in a state waiver allowing the use of vouchers at farmers markets. The objective of this study was to evaluate motivations and barriers to participation among vendors and markets and explore WIC staff experience in promoting farmers markets.

## Methods

### Study design

We used mixed methods to evaluate the program after implementation (Table 1). We examined project records to determine redemption rates of WIC vouchers and SNAP benefits at intervention markets. We also surveyed or interviewed market managers and vendors about their decision to accept or decline vouchers or SNAP or both and vendors’ employees about their experience accepting SNAP and vouchers. We surveyed WIC clinic staff and conducted a focus group with WIC staff and market managers to evaluate training needs for conducting effective client outreach. The University of Washington’s Human Subjects Division deemed our study exempt.

**Table 1:** Timetable of Intervention and Evaluation Activities of Farmers Market Access Project, King County, Washington, 2010–2012

Activities	2010									2011																	2012				
	J		A	S		O		N	D	J	F	M			A	M	J	J	A	S		O	N		D		J		F		M
**Intervention**																															
Advisory Committee forms, meets monthly	■	■		■	■		■		■	■	■		■	■		■	■	■	■	■		■	■		■		■		■	■	
SNAP and WIC voucher workshops for vendors and market managers		■							■																						
Outreach newsletters and e-mails distributed to vendors									■	■	■																				
WIC voucher training sessions for vendors														■		■	■	■													
SNAP and WIC client outreach materials developed and distributed to WIC clinics														■		■	■	■	■												
Markets open, markets and vendors accept SNAP/EBT and credit/debit																■	■	■	■	■		■									
Vendors accepting WIC vouchers (1 month after markets opened due to WIC approval of voucher applications)																	■	■	■	■		■									
Outreach (signs and ethnic media) to SNAP and WIC clients																	■	■	■	■											
**Evaluation**																															
Managers interviewed and vendor stall employees surveyed at markets																			■		■										
WIC staff surveyed in clinics																						■									
Focus group with WIC staff and market managers																								■							
Telephone and e-mail survey of vendors																										■		■	■		

Abbreviations: SNAP, Supplemental Nutrition Assistance Program; WIC, Special Supplemental Nutrition Program for Women, Infants, and Children; EBT, electronic benefits transfer.

### Intervention

FMAP recruited all 9 farmers markets in the CPPW target region of South King County for the pilot. FMAP offered 2 options for these markets to accept SNAP. Option 1: Markets, through their governing body and manager, could become authorized SNAP retailers and either purchase or lease a wireless EBT/credit/debit terminal. FMAP reimbursed a year’s operating costs, excluding credit/debit transaction fees, plus the cost of a 1-year lease or half the purchase price. One terminal can serve an entire market, meaning shoppers swipe their EBT or credit/debit cards at the market entrance in exchange for dollar “tokens” to be used at vendors’ stalls. Three markets were authorized SNAP retailers before the intervention, although 2 lacked terminals, and one could not accept credit/debit. FMAP offered them EBT/credit/debit terminals. Option 2: FMAP reimbursed individual vendors for a 1-year terminal lease or 50% of purchase cost plus a year’s operating costs, excluding credit/debit transaction fees, in exchange for becoming authorized SNAP retailers. Vendors who received terminals could use them to accept SNAP and credit/debit at any retail location where they sold, including at markets that received a market-wide terminal. Vendors were eligible to apply for a terminal if they had a stall at an intervention market and sold at least 50% SNAP-eligible products, as determined by USDA.

For this pilot, vendors at the intervention markets could apply to Washington State for authorization to accept WIC vouchers. To expedite the contracting process, the state limited eligibility to vendors already participating in an existing WIC program. Vendors were required to complete an application and attend a training session. When accepting vouchers, sales staff were required to check the shopper’s identification, obtain a signature, and record purchase information.

The first 10 months of the intervention were dedicated to planning and outreach. The FMAP coordinator consulted with an FMAP advisory committee of WIC and SNAP program staff, vendors, and market representatives to recruit and train market managers and vendors. An advisory committee member met with and e-mailed WIC clinic lead staff to explain the project and encouraged them to educate their staff and clients. The advisory committee also produced and translated outreach materials for distribution at WIC clinics and markets.

### Setting

Our study was conducted from August 2011 through February 2012 — beginning 3 months after markets opened until 4 months after markets closed — to gather feedback at the height of the sales season and to allow vendors to reflect on their experience after markets closed. The participating markets were located in an area encompassing 44% of the county’s 1.9 million residents (8). This area is more racially diverse and has higher rates of poverty than the rest of the county (8). Of residents in this region, 20% receive SNAP benefits and 3% receive WIC benefits, compared with the county as a whole, at 6% and 2%, respectively. The only food deserts in King County are in this region (9).

### Study population and data collection

Participation in the intervention was determined by asking market managers to provide estimates of the number of vendor stalls and SNAP-eligible vendors at their markets and reviewing FMAP records. FMAP records also provided information on redemption rates for SNAP and WIC vouchers. For surveys and interviews, we had 3 study groups: vendors, market managers, and WIC clinic staff (Figure). All participants had to be 18 years of age or older, speak English or Spanish, or have someone to interpret, and agree to participate in the study. The FMAP committee consulted on survey and focus group questions and the interview guide (Appendix), which were then reviewed by public health evaluators and pilot tested with the target audience.

**Figure:**
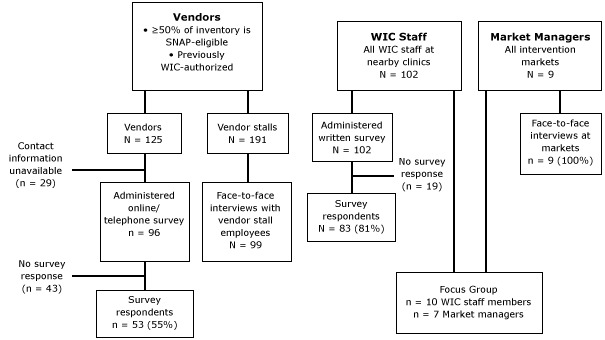
Eligibility criteria and sample sizes for vendors, market managers, and WIC clinic staff included in the evaluation of the Farmers Market Access Project, King County, Washington, 2011. N = the number surveyed or invited to be surveyed; n = number responding. Abbreviations: WIC, Special Supplemental Nutrition Program for Women, Infants, and Children; SNAP, Supplemental Nutrition Assistance Program; FMAP, Farmers Market Access Project.

Vendors eligible to apply to accept SNAP or vouchers and for whom we had accurate contact information were surveyed 2 to 4 months after markets closed. This Internet and telephone survey asked vendors about factors influencing their decision to participate in FMAP. We sent 2 e-mails and made 1 telephone call to each nonrespondent. 

We also surveyed employees at market stalls who were either participating in FMAP or were selling at a market that had received a terminal through FMAP. We asked about their experience accepting vouchers and SNAP at the market where they were operating that day. All markets operated 1 day per week, and we visited each for 1 full day beginning 3 months after markets opened.

Using a semistructured interview guide, we interviewed managers at each market during our market visit. Because they influenced decisions made by the market’s governing organization, we asked about triggers and barriers to installing a central market EBT terminal through FMAP and post-intervention intentions to maintain a terminal. 

We surveyed all WIC staff at 13 clinics near the markets during the month in which markets closed through a self-administered questionnaire. Lead staff distributed and collected the surveys at a staff meeting.

We convened a focus group 1 month after markets closed. Managers from each of the markets and 1 representative from each of the WIC clinics were asked to participate. Advisory committee members from both WIC and market management facilitated the focus group.

### Data analysis

We described quantitative data by using frequencies calculated with PASW Statistics 18.0 (SPSS Inc, Chicago, Illinois). We reported Likert scale responses as mean and standard deviation (SD). The focus group discussion was audio-recorded and transcribed. One researcher coded the focus group, open-ended survey items, and interview responses, and formed themes and key examples. The second and third authors reviewed this analysis, iteratively discussing disparate interpretations.

## Results

### Vendor acceptance of SNAP and WIC vouchers

Before the intervention, 3 markets representing a total of approximately 80 vendor stalls accepted SNAP. Through FMAP, 6 of the 9 markets either received terminals or upgraded their existing equipment. Of the 125 vendors eligible to receive a terminal, 10 (8%) became SNAP retailers and either received a terminal or upgraded their credit/debit terminal to accept SNAP. FMAP also permitted 63 additional vendor stalls to accept SNAP, an increase of 79% from 80 to 143 stalls.

Of the 88 vendors eligible to apply to accept vouchers, 38 (43%) applied and were authorized; 25 of these (66%) successfully redeemed vouchers. Pregnant and postpartum WIC clients receive $10 in vouchers monthly, plus $6 per child aged 1 to 5 years. Vendors accepted vouchers from July through October 2011. Of 95,244 vouchers distributed at area WIC clinics during this period, 427 (0.4%) were redeemed at the FMAP farmers markets, totaling $3,052; traditional retailers in the region redeemed $582,000 in vouchers during this period.

### Vendors and employees

#### WIC Vouchers

The most common reason cited for not applying to accept vouchers was an inability to attend the required training (Table 2). One vendor explained, “I need to be able to sign up before the market season begins because I can’t handle both [selling and attending a training] at once.” Some respondents learned of the project too late to apply or thought accepting or depositing vouchers seemed too complicated.

**Table 2:** Internet and Telephone Survey of Vendors (N = 53) Eligible to Participate in Farmers Market Access Project, 9 Farmers Markets, King County, Washington, 2011

Factor (No. of Respondents)	n
**WIC Cash Value Vouchers**	
**Nonparticipating vendors (n = 19)^a^**	
Reasons for not participating^b^	
Unaware of project	6
Unable to attend required training	8
Accepting or depositing vouchers too complicated	5
Did not seem profitable	2
Other^c^	4
Interested in applying to participate next year	
Yes	4
No	6
Maybe/only if there were changes	9
**Participating vendors (n = 31)^a^**	
Reasons for participating^b^	
To make it easier for WIC customers to buy my food	27
I felt like I would earn more money	15
Other^c^	5
Interested in participating again next year^d^	
Yes	22
No	5
Maybe	2
**Wireless EBT/Credit/Debit Terminals for Vendors**	
**Nonparticipating vendors (n = 43)^a^**	
Reasons for not participating^b^	
Did not want to pay credit/debit fees	19
Did not seem profitable	14
I mostly sell at markets that have their own terminal	9
Application was too complicated	6
Unaware of project	6
Project launched during market season	5
Accounting and usage seemed too difficult	5
Other^c^	5
Interested in applying for a terminal next year	
Yes	6
No	25
Maybe	12
**Participating vendors (n = 9)^a^**	
Reason for participating^b^	
To make it easier for SNAP clients to buy my food	6
To increase sales	6
Other^c^	4
Do you feel it was worth your time and effort to get a terminal?^e^	
Yes	6
Unsure	2
No	0
Plan to continue using terminal next year^e^	
Yes, even without financial assistance	6
Yes, but only with financial assistance	1
No	1

Abbreviations: WIC, Special Supplemental Nutrition Program for Women, Infants and Children SNAP, Supplemental Nutrition Assistance Program.

a 53 vendors were surveyed; 3 were not eligible to accept SNAP and thus did not complete this portion of the survey. One vendor who was eligible for SNAP and cash value vouchers completed only the portion of the survey that addressed vouchers.

b Participants were given 10 responses to choose from and allowed to choose up to 2 reasons.

c “Other” includes both the open response “other” and responses chosen by only 1 participant.

d Two participants did not answer the question.

e One participant did not answer the question.

Most participating vendors reported participating because they wanted to make it easier for WIC clients to purchase their food. One vendor explained, “The process was a little different because you had to write things down, but other than that, it would just be one more way of letting them have my food.” Nearly half said they accepted vouchers to earn more money, and most were interested in accepting vouchers again in the future.

Forty-seven of the 99 employees surveyed at markets accepted vouchers. Most experienced no problems, but 12 reported difficulty following the steps required in voucher transactions. Noting that banks would not redeem the vouchers without proper transaction documentation, 1 vendor lamented, “We’re so busy that we can’t stop and check IDs. We only got $100 in [vouchers] and every one of them was filled out wrong, so it was a total loss.”

#### Supplemental Nutrition Assistance Program

Among the 43 vendors who did not receive a terminal, the most common reason for nonparticipation was the expense of credit/debit sales fees (Table 2). The second most common response was the belief that the terminal would not be profitable; half of these respondents explained they sold at few markets and/or had total sales too low to warrant accepting noncash currencies. Six of the nonparticipating vendors expressed interest in future participation.

Among the 9 vendors surveyed who acquired a terminal, the most common reasons for doing so were to make it easier for SNAP clients to buy their food and to increase sales. When asked if they felt it was worth their time and effort to get a terminal, 6 of 9 said yes. Two vendors were unsure, noting low SNAP profits: “EBT sales at farmers markets were low, but I believe they will grow with time.” One vendor said he would not continue using his terminal without a subsidy.

Of the 16 employees who operated terminals, 7 said the terminal was slow to process cards. One vendor had never gotten his terminal to work successfully.

Of the 99 employees surveyed, 82 sold at markets that received a market terminal. Fifty-two of these said the market’s SNAP/credit/debit capabilities increased their stall’s profits at that market. Most wanted the market to continue the system.

### Market managers

Three market managers operated city- or volunteer-run markets and chose not to get a terminal. These managers expressed concern over the legal responsibilities associated with becoming a SNAP retailer and the additional accounting work in handling SNAP sales and reimbursement. In contrast, 5 of the 6 participating markets were run by independent organizations with paid staff and the capacity to absorb additional work. These managers cited increased convenience and a larger customer base as their primary motivations. However, 1 manager stated, “From a business perspective, it’s a lot of overhead for very little money. It’s an access issue — we wanted to reach out.” Two managers said they had been hesitant about the extra responsibility of accepting SNAP, noting that they would not have participated without FMAP’s encouragement. Despite some initial reluctance, all managers were happy with their improved SNAP/credit/debit capabilities and planned to retain their terminal without subsidies.

### WIC clinic staff

The mean rating of FMAP knowledge was high among the 83 WIC staff surveyed, but fewer than half reported discussing with every client opportunities to use vouchers or SNAP at farmers markets (Table 3). Most who did indicated that clients responded enthusiastically or wanted more information about using benefits at markets. Some, however, said clients with limited English proficiency were confused or overwhelmed by this information. Respondents cited transportation/market location and language as the most common barriers to using vouchers at markets.

**Table 3:** Survey of WIC Staff (N = 83) on Knowledge of Farmers Market Access Project, Outreach to Clients, and Perceptions of Client Interest in Shopping at Farmers Markets, King County, Washington, 2011

Topic/Factor (No. of Respondents)	Value^a^
**Farmers Market Access Project outreach (n = 83)**	
Frequency of discussion of WIC voucher opportunities at markets per client visit (n = 83)	
At least once to more than once with every client	37 (44.6)
With many, but not all, clients	32 (38.6)
Rarely or never	14 (16.9)
Frequency of discussion of SNAP opportunities at markets per client visit (n = 82)	
At least once with every client	25 (30.5)
With some, but not all, clients	38 (46.3)
Rarely or never	19 (23.2)
**Staff perceptions of client interest in Farmers Market Access Project^b^^, ^ c**	
English-speaking clients’ response to learning about WIC voucher opportunities (n = 64)	
Enthusiastic/wanted more information	53 (82.8)
Confused/overwhelmed	3 (4.7)
Indifferent/uninterested	9 (14.1)
Limited-English–proficiency clients’ response to learning about WIC voucher opportunities (n = 64)	
Enthusiastic/wanted more information	50 (78.1)
Confused/overwhelmed	12 (18.8)
Indifferent/uninterested	9 (14.1)
English-speaking clients’ response to learning about SNAP opportunities (n = 46)	
Enthusiastic/wanted more information	36 (78.3)
Confused/overwhelmed	5 (10.9)
Indifferent/uninterested	9 (19.6)
Limited-English–proficiency clients’ response to learning about SNAP opportunities (n = 49)	
Enthusiastic/wanted more information	35 (71.4)
Confused/overwhelmed	11 (22.4)
Indifferent/uninterested	7 (14.3)
**Staff perceptions of client barriers^c^^, ^ d**	
Barriers clients face to using WIC vouchers at markets (n = 66)	
Transportation/location	36 (54.5)
Language	21 (31.8)
Schedule	11 (16.7)
Lack of participating vendors or markets	10 (15.2)
**WIC staff knowledge of Farmers Market Access Project^e^** **(n = 80)**	
Mean (SD)	7.4 (2.2)

Abbreviations: WIC, Special Supplemental Nutrition Program for Women, Infants, and Children; SNAP, Supplemental Nutrition Assistance Program; SD, standard deviation.

a All values are numbers (percentages), unless otherwise indicated.

b Excludes respondents who indicated promoting the Farmers Market Access Project rarely or never (n = 14 for WIC vouchers; n = 19 for SNAP).

c Respondents allowed to choose up to 3 responses; responses do not total 100%.

d Open-response question; answers coded and 4 most common responses reported; responses do not total 100%.

e Knowledge measured according to 10-point Likert scale (1 = very low; 10 = very high).

Many of the themes identified by the focus group comprising market managers and WIC staff supported our WIC staff survey results. Both parties felt better coordination between WIC and markets would improve outreach to shoppers. One WIC staff member said it was difficult to tell clients about the market because she had never been. She suggested, “We could set up a day to have a tour for all the WIC staff, how it’s available and how it runs. Recruit some of our clients to help other clients at the market.” Another WIC staff member agreed: “We had to explain to clients how to use the [vouchers] and the EBT, but we really weren’t connected with the farmers; we did not know how the process was going to be.”

As the focus group concluded, WIC staff and market managers suggested future collaboration: “You could attend one of our staff meetings and talk about how we can do a better job of promoting the market,” a WIC staff member said to a manager. “I’m going to make sure we have a WIC person at our market,” resolved a manager.

## Discussion

We found that farmers markets and vendors wanted to encourage shoppers using WIC and SNAP benefits and, when offered support through FMAP, were willing to take on some inconvenience to serve them. This expands on conclusions from a 2003 study on the use of vouchers in the Senior Farmers Market Nutrition Program, which found vendors would make accommodations to participate in programs serving low-income seniors (10). That said, the ongoing costs associated with terminals and difficulties securing voucher authorization and redemption were barriers. Our findings suggest that vendor participation could be increased by simplifying the enrollment and transaction processes and reducing costs associated with accepting benefits. Other communities have subsidized costs and have seen increases in market participation and shopper use (11–13).

Few vendors chose to get their own terminals, and most vendors viewed FMAP’s short-term subsidies as insufficient to offset terminal operation costs, consistent with a previous study (14). In contrast, the market-wide terminal model offered an economy of scale, providing lower cost relative to terminals operated by individual vendors. Other studies cite the pros and cons of both models, reporting an accounting burden on market managers as SNAP usage increases and, in one intervention, higher total SNAP sales when vendors had their own terminals compared with the market-wide terminal model (13,14). Because of the advantages and disadvantages of each model, as well as our finding that some markets are unlikely to adopt terminals, we recommend that both models be encouraged.

SNAP and WIC voucher redemption rates during the intervention were low, a problem faced by many farmers markets (13,15). The nationwide increase in SNAP spending at markets as the number of markets accepting SNAP has increased suggests that redemption rates grow as markets and vendors perceive that opportunity costs are acceptable and shoppers’ awareness increases (12). Previous research cites market inaccessibility (eg, transportation and hours), unfamiliarity with farmers markets, and a perception of higher prices as barriers to attracting low-income shoppers (13,15,16). Our findings suggest that interventions to address these barriers — and robust outreach — are needed for a supply-side intervention to succeed. Relationship building between WIC staff and market personnel is one way to encourage outreach, because WIC staff may be motivated to promote farmers markets when they feel connected to and knowledgeable about them. Other communities have succeeded in attracting low-income shoppers and increasing benefit redemptions at markets through strong community and organizational partnerships (17,18). In addition, incentives for SNAP and WIC shoppers, such as free vouchers and matching funds for spending benefits at markets, have been successful at attracting low-income shoppers (13,19).

Our study has several limitations. Although we included the views and experiences of multiple stakeholders, we surveyed a small number of cases — those in FMAP. These markets all serve low-income areas; our findings may not be representative of farmers markets in general. Because we only surveyed English and Spanish speakers, some vendors were not represented. We had a low response rate among employees because some stalls were not present on the day of our visit and because of time and language barriers. 

Our study suggests that, when given support, farmers markets will accept some inconvenience to serve nutrition assistance beneficiaries; however, without subsidies, many will find the costs of equipment and fees too high. In addition, market managers and WIC staff are willing to work together to improve outreach to low-income shoppers. Although nutrition assistance programs align with the social values of many farmers markets and their vendors, these programs as currently designed are better suited to the capacity of large food retailers. By translating pilot interventions such as this into wider-reaching policies, adapting the requirements of federal nutrition assistance programs to meet the capabilities of farmers markets, and providing incentives for the use of nutrition benefits at markets, policy makers have the opportunity to shift the local food environment toward greater access to fresh produce for lower-income residents.

## Appendix: Survey, Interview, and Focus Group Guides

**A. Vendor Survey Questions:**

A1. Special Supplemental Nutrition Program for Women, Infants, and Children (WIC) cash value voucher non-participants:

(WIC vouchers were commonly known by vendors as “$6 and $10 WIC Fruit & Vegetable Checks,” and thus this term was used in the vendor survey.)

This year, there was a project that would allow vendors to accept $6 and $10 WIC Fruit & Vegetable checks. Why did you decide not to participate? (You may choose up to 2 answers.)

- I didn’t know about the project
- I don't sell the right kind of food
- I couldn’t attend the required training
- The application process seemed too complicated
- It didn’t seem like I would make much money from it
- The check amounts were too large
- I was worried it would be hard to deposit the checks and I would lose money
- The penalties for making a mistake when depositing a check were too high
- It is too hard to accept checks from customers
- I, or my employees, don’t speak enough English
- Other (please specify):

Do you want to accept the $6 and $10 WIC Fruit & Vegetable checks next year? (Please choose only 1 answer)

- No
- Maybe, I need more information
- Yes

A2. WIC Cash Value Voucher Participants:

Why did you decide to apply to accept $6 and $10 WIC Fruit & Vegetable checks? (You may choose up to 2 answers.)

- I wanted to make it easier for WIC customers to buy my food
- I felt like I would earn more money
- The process seemed easy
- Other (please explain):

Do you plan to continue to accept $6 and $10 WIC Fruit & Vegetable checks next year? (Please choose only 1 answer.)

- Yes
- Maybe
- No

A3. SNAP/EBT Nonparticipants:

This year, there was a project helping vendors get a wireless EBT (food stamps)/credit/debit terminal if they applied to accept EBT (get an FNS number to be a SNAP retailer). Why did you decide not to get a wireless terminal? (You may choose up to 2 answers.)

- I didn’t know about the project
- I didn’t think I would make much money from it
- The application was too complicated
- The accounting seemed like too much work
- Too much risk if people made mistakes
- I mostly sell at markets with their own terminals
- I didn’t want to pay credit/debit fees
- I, or my employees, don’t speak enough English
- Other (please explain):

Do you want to apply for a wireless terminal next year?

- No
- Maybe, I need more information
- Yes, I’m interested

A4. SNAP/EBT Participants

Why did you decide to get a wireless terminal through the Farmers Market Access Project? (You may choose up to 2 answers.)

- To make it easier for people who have EBT (food stamps) to buy my food
- To increase sales
- To make it easier for people shopping with credit/debit
- It seemed like a good opportunity to get a cheap wireless terminal
- I wanted to use the terminal at other locations (CSA, farm stand, etc)
- Other (please explain):

Did you feel it was worth your time and effort to get set up to accept EBT and credit/debit?  (Please choose only 1 answer.)

- Yes, it was worth it
- No, it was not worth it
- I’m not sure

Do you want to continue using the wireless terminal next year? (Please choose only 1 answer.)

- Yes, I’ll continue even without financial support
- Yes, but only if there is some financial support
- No, I don’t want to continue using the terminal
- No, because the markets I sell at already have their own terminal
- Unsure

A5. For all vendors, regardless of participation:

Is there anything else you’d like to add about the wireless terminal project or the $6 and $10 WIC Fruit & Vegetable checks? (Optional) **(Open response)**

**B. Vendor Stall Employee Survey Questions**

Does this stall have a wireless terminal that is capable of accepting EBT/food stamps? (If yes) Have you experienced any problems accepting EBT? Please describe.

Does this stall accept $6 and $10 WIC Fruit & Vegetable checks? (If yes) Have you experienced any problems accepting $6 and $10 WIC Fruit & Vegetable checks? Please describe.

(For employees at markets with a central market SNAP/credit/debit terminal): Do you want this market to continue using the SNAP/credit/debit token system next year?

**C. WIC Staff Survey Questions**

How often during WIC visits do you discuss opportunities to use cash value vouchers at farmers markets with your clients? (Please choose only 1 answer.)

- More than once with every client
- At least once with every client
- With most, but not all clients
- With about half my clients
- Rarely

Do you talk with your clients about opportunities to use SNAP/EBT benefits at farmers markets? (Please choose only 1 answer.)

- Yes, with every client
- Yes, with every client I know receives SNAP/EBT
- With most, but not all clients
- Only if they ask
- No, I rarely or ever talk to my clients about EBT at farmers markets

How do the majority of your English-speaking clients respond to learning about WIC cash value voucher opportunities at farmers markets? (You may choose up to 2 answers.)

- Enthusiastic
- Curious — want more information
- Overwhelmed
- Indifferent
- Confused
- Uninterested

How do the majority of your non-English-speaking clients respond to learning about WIC cash value vouchers opportunities at farmers markets? (You may choose up to 2 answers.)

- Enthusiastic
- Curious — want more information
- Overwhelmed
- Indifferent
- Confused
- Uninterested

If you talk to many of your clients about EBT opportunities at farmers markets, how do the majority of your English-speaking clients respond? (You may choose up to 2 answers.)

- Enthusiastic
- Curious — want more information
- Overwhelmed
- Indifferent
- Confused
- Uninterested

If you talk to many of your clients about EBT opportunities at farmers markets, how do the majority of your non-English-speaking clients respond? (You may choose up to 2 answers.)

- Enthusiastic
- Curious — want more information
- Overwhelmed
- Indifferent
- Confused
- Uninterested

On a scale of 1 to 10, how knowledgeable do you feel about the Farmers Market Access Project (FMAP)?

(not at all knowledgeable) 1 2 3 4 5 6 7 8 9 10 (very knowledgeable)

What barriers do your clients face in using WIC Fruit & Vegetable checks at farmers markets? (open response question)

**D. WIC Staff and Market Manager Focus Group Guide**

What went well with the Farmers Market Access Project?

What should be improved?

How has this experience changed your perception of the WIC staff/market managers?

How did communication between markets and WIC staff work? What worked? What needs to be improved?

What can the markets do to better attract WIC clients?

What would you like to see WIC staff and market managers do differently?

What could you do to make collaboration between WIC clinics and markets better in the future?

**E. Market Manager Interview Guide**

Did you receive a wireless EBT/credit/debit terminal through the Farmers Market Access Project?

What were the reasons for your choice?

If you don’t have a wireless EBT/credit/debit terminal, do you plan to get one next year? Please explain why.

If you do have a wireless EBT/credit/debit terminal, do you plan to keep it for next year even if subsidies are no longer provided? Please explain why.

Anything else you’d like to add?
